# The biogenetical revolution of the Council of Europe - twenty years of the Convention on Human Rights and Biomedicine (Oviedo Convention)

**DOI:** 10.1186/s40504-018-0073-2

**Published:** 2018-05-16

**Authors:** Oktawian Nawrot

**Affiliations:** Department of Theory and Philosophy of Law and State, Faculty of Law and Administration, University of Gdansk, ul. Jana Bażyńskiego 6, 80-309 Gdańsk, Poland

**Keywords:** Human dignity, Human biogenesis and human rights, Legal protection of human embryo, The Council of Europe, The biomedicine convention

## Abstract

The Council of Europe’s legal regulation concerning development of biology and medicine undoubtedly form the most interesting, but certainly not perfect, over-national system of protection of human beings in prenatal stages of development. The strength of the mentioned system is that it based on well-known and common acceptable values and rules such as human dignity and its protection. The aim of the paper is to present the reasons behind adopting such a system, as well as the consequences of the latter.

The author argues that in such a way a revolution within the human rights system of the Council of Europe took place. This revolution caused a significant expansion of the Council of Europe’s system of human rights’ protection and changed the perspective of the protection from vertical to the horizontal.

Summary: 1. The Temple Rebuilt – 2. Dignity as the basis of bioethical regulations – 3. The identity of the human being – biological reductionism or a new basis for metaphysics?

## Introduction

In conclusion, published in 1990, in his post-doctoral thesis entitled ‘The Law in view of intervention in the nature of human procreation’, Marek Safjan stated that: “The conflict in the field of fundamental values of axiological assumptions rarely reveals itself with such clarity as it has in this book on the subject of legal problems occurring against the backdrop of the achievements of contemporary biogenetics. This was not just about an answer to a question of whether law, in its current form, either is, or is not, ready to resolve entirely new and never before seen or resolved problems in the legal tradition. Moreover, the question, in the light of the analysis, is a rhetorical one: of course, the law cannot deal with most of these issues alone. The question really comes down to whether the law, given the bitterness of the conflict and the dramatic contrast of the arguments being put forward, is really in a position to find its own solutions in this regard whilst not becoming entangled in deep contradiction and not dismissing those fundamental, basic values which were at the root of the regulations in question and which are the result of centuries of tradition and of evolution” (Safjan 1990). This particular doubt, after 20 or so years, and despite significant changes in both domestic and international legal systems, nevertheless retains its topicality; it even appears to gain in pertinence in the light of advances in the biomedical sciences.

This conflict, in relation to the biotechnological revolution taking place before our eyes, is playing out directly into the line of humanity – humans are, in fact, dealing here with paradigms of law and also becoming the axiological *basis* of it. On the one hand, biomedicine invites a vision of a world in which: “the blind receive sight, the lame walk, those who have leprosy are cleansed, the deaf hear, the dead are raised” (Matthew 11:5), and in exchange – not only for a seemingly fair price for this kind of investment in a ‘brave new world’ – but in exchange for unhindered development. On the other hand, the instrumentalisation of human beings as such – being reduced to the purely biological – has never seemed so real as it does now. Of course, history provides us with all too many cases of heinous instances of the objectivization of individuals, societies and even entire nations, but never has it been the case that instrumentalisation has been a potential threat to *every* human being. Furthermore, the cause of instrumentalization was never being a human being – belonging to the *Homo sapiens* species. Currently, the price that must be paid for maintaining the pace of progress in biomedical science and techniques seems to be this simple, yet simultaneously an extremely significant and consequential step – the sacrificing of the human being, in the earliest stages of its development, for the good of humanity (Honnefelder 2005).

## The temple rebuilt

Outlined above, in perhaps a little too much of a gloomy tone, this conflict does, however, require fundamental decisions on an axiological plane. Finally, this is not about the biolaw system meeting the requirements set out in the framework of legal positivism – contemporary legislators are undoubtedly capable of coming to grips with this – but about its morality. Thus, the central question is this: In what direction should biolaw attempt to develop? Which values should be considered as fundamental, and which as secondary? How are we to solve the conflicts arising between these values? (Safjan 2007).

The response of the Council of Europe to the question raised above is, essentially, the first which takes this on, on such a large scale, to establish standards with regard to biomedical intervention associated with human biogenesis. Before assessing this, it is necessary to underline that such an attempt regarding regulations on such issues goes beyond the traditional zone of proposals or principles of a deontological nature. Besides the recommendations and the resolutions of the Parliamentary Assembly and the Committee of Ministers of the Council of Europe, the Convention on Human Rights and Biomedicine and the Additional Protocols are also on this very topic. The ratification of these documents requires, from Member States, specific obligations. Moreover, in accordance with Article 23 of the Biomedicine Convention, States Parties to the Convention have to provide appropriate judicial protection to prevent or to put a stop to unlawful infringement of the rights and principles set forth in the Convention (Para. 140 Explanatory Report to the Convention for the protection of Human Rights and Dignity of the Human Being with regard to the Application of Biology and Medicine: Convention on Human Rights and Biomedicine).

The effectiveness of this protection is further influenced, and this results from Article 25 of the Biomedicine Convention, by the obligation of the Parties to guarantee the application of appropriate sanctions in the event of a violation of the provisions of the Convention. As explained by the Steering Committee on Bioethics, these sanctions should, above all, meet the criteria of necessity and proportionality. Setting them up, national legislature should, in the first instance, bear in mind the contents and meaning of the provisions of the Treaty which could be violated, as well as the gravity and possible consequences of this violation – both on an individual and social basis (Para. 148 Explanatory Report to the Convention for the protection of Human Rights and Dignity of the Human Being with regard to the Application of Biology and Medicine: Convention on Human Rights and Biomedicine). Moreover, on the basis of Article 24 of the Biomedicine Convention, a person who has suffered undue damage resulting from an intervention is entitled to fair compensation.

Turning to an assessment of the biogenetic standards of the Council of Europe, we should recall that these fit into the much broader system of protection of human rights and freedoms of the Council of Europe, which is a part of this system’s development. What directly demonstrates this is the term that appears in the title of the Treaty: ‘protection of human rights’. As underlined by the CDBI (Steering Committee on Bioethics) in its Explanatory Report, this is directly tied in with the principles honoured by the Convention for the Protection of Human Rights and Fundamental Freedoms of 4th November 1950 (Para. 9 Explanatory Report to the Convention for the protection of Human Rights and Dignity of the Human Being with regard to the Application of Biology and Medicine: Convention on Human Rights and Biomedicine). Consequently, one should accept that the provisions of the Convention on Human Rights and Biomedicine are in keeping with the provisions – as well as the philosophy – adopted in the framework of the Convention for the Protection of Human Rights and Fundamental Freedoms. Acts which are mentioned in the preamble of the Convention on Human Rights and Biomedicine must also lead us to similar conclusions as this. These acts make up a kind of foundation for all the bioethical regulations, including the biogenetic regulations, of the Council of Europe. From a philosophical point of view, this type of positioning of bioethical issues can be seen as the task of the Council of Europe in taking a strong position in the ongoing dispute on the importance of bioethical regulations. The claim that the bioethical problem, including the issue of human biogenesis, is located in the domain of human rights, entails serious moral and legal consequences. Quite apart from the concrete solutions adopted by the Council of Europe, it is necessary to highlight the fact the list of values which it has been decided must be protected, now moves up to the level of universal values.

This type of positioning of the bioethical problem, in particular of bioethics, evokes numerous reservations on the part of some of those who participate in bioethical discourse. First of all, one may argue that the regularisation of bioethics in the domain of human rights ignores philosophical differences, as expressed in, amongst others, the diversity of attitudes towards the use of biomedicine’s achievements, as well as research performed in this field (CAHBI – Ad hoc Committee of experts on Bioethics 24–27/03/92). One then notes that the extension of the domain of human rights in view of the biogenetic problem is just a visible measure which, in fact, does not introduce any actual changes. Systems of the protection of human rights, including that of the Council of Europe, shaped their outlines at a time when the bioethical problem basically remained outside the sphere of interest of legislators. Therefore, their characteristic feature is their ‘person-centeredness’ – through the prism of the “person” will assess violations of rights and freedoms (Freeman 2002; Morsink 2000). This hypothesis is partially confirmed by the widespread occurrence within the framework of the biogenetic regulations of the Council of Europe of the term ‘human being’, which is used alongside the term ‘person’, and is often understood as being a synonym of the word ‘human’. Consequently, the scope of people’s rights and freedoms does, in no case, change (Para. 9 Explanatory Report to the Convention for the protection of Human Rights and Dignity of the Human Being with regard to the Application of Biology and Medicine: Convention on Human Rights and Biomedicine; X v. Norway; X v. Austria; Brüggemann and Scheuten v. Germany; X v. the United Kingdom; Open Door and Dublin Well Woman v. Ireland; Reeve v. the United Kingdom; Boso v. Italy). Furthermore, one notes that the regularisation of bioethics in pre-existing systems of the protection of human rights could lead to many ambiguities and even intra-system contradictions. This above all applies to situations when new regulations employ vague terminologies (Hottois 2000).

The above reservations should therefore be considered as being, to a certain extent, mistaken. The allegations rejected by the Council of Europe on the diversity of the philosophical sides of the bioethical discourse does not coincide with the contents of the legal acts of the Council of Europe. In many of these, as well as in matters of fundamental importance, and pursuant to the nature of the given act, the Member States of the Council of Europe and possibly the Parties to the Convention or Protocol, have left room for the interpretation of individual provisions. As an example the States Parties to the Bioethics Convention may use a margin of appreciation in matters of the connotations and denotations of the terms ‘everyone’/‘person’ and ‘human being’(CDBI 6–9/07/93). Similarly, and in accordance with the comments of the Steering Committee on Bioethics contained in the Explanatory Report to the Additional Protocol to the Convention for the Protection of Human Rights and Dignity of the Human Being with regard to the Application of Biology and Medicine on the Prohibition of Cloning Human Beings, the Parties retain the possibility to define the scope of the expression ‘human being’ for the purposes of the application of the Protocol (Para. 6 Explanatory Report to the Additional Protocol to the Convention for the Protection of Human Rights and Dignity of the Human Being with regard to the Application of Biology and Medicine, on the Prohibition of Cloning Human Beings). Often, also, the actual scope of the obligations imposed on the State depends on the legal solutions adopted by them at a level of national legislation. Emphatic proof of the above thesis is the oft-criticised Article 18, paragraph 1 of the Biomedicine Convention, which states that for countries in which the law allows research on in vitro embryos it will ensure the adequate protection of the embryo. Consequently, the allegation of philosophical authoritarianism against the Council of Europe in the field of bioethics, is totally inconsequent. Contrarily, it would appear that, to a certain extent, these regulations ‘err’ into excessive pluralism.

The fact that the Council of Europe’s bioethical regulations include, and most certainly perceive, philosophical differences in particular systems of values working in parallel, does not mean that they do not distinguish between these. The axiological neutrality of law is not only *not* possible, but it is also undesirable. Every legislator must, at the outset, define the values which he or she deems worthy of protection. In such a way did the United Nations act on 10th December 1948, in their ground-breaking text entitled ‘The Universal Declaration of Human Rights’. And in a similar way did the creators of The Convention for the Protection of Human Rights and Fundamental Freedoms proceed also. And once again, in a similar way did the creators of the bioethical standards of the Council of Europe proceed. Allegations of the identification, within the framework of the bioethical regulations, of a specific recognised axiology shows, *in abstracto*, a total misunderstanding of the nature, character and the objectives of law. In turn, this allegation, understood as the arbitrary preference of a specific axiological system, does not take into account the real shape of the bioethical standards of the Council of Europe – as mentioned in the above paragraph – or their conditions and their ways of seeking redress.

The ostensibility allegation introduced by the problem of bioethics into the area of the regulations on human rights comes directly up against the facts here. It is undoubtedly true that the foundations of the Council of Europe’s system of human rights’ protection happened at a period when the bioethical problems pertained more to the domain of science-fiction than to that of reality. However, this system, and in particular its flagship document – ‘The Convention for the Protection of Human Rights and Fundamental Freedoms’ – is a living instrument (Tyrer v. the United Kingdom), now in operation for 60 years. Keeping it ‘alive’ has become possible, amongst other things, because of a departure from its original creators’ ‘initial intentions’, whilst still upholding its underlying ‘spirit’. Thanks to this, the European Court of Human Rights is in a position to resolve effectively bioethical disputes. In this light, as well, one can discern a bold attempt to develop bioethical standards. The Council of Europe’s system for the protection of human rights is constantly evolving, and adapting to changing realities and at the same time modifying them.

The introduction of the problem of human biogenesis can be seen not only as an act having nothing whatsoever to do with ostensibility, but even the contrary – as a kind of revolution happening within the framework of The Council of Europe’s system for the protection of human rights. Regardless of above reservations, this kind of positioning of the analysed problem places it amongst the fundamental issues facing democratic states of law. At the same time, its importance calls – as was the case 65 years ago with the protection of human rights and freedoms – for the creation of specific supranational protection mechanisms. Furthermore, the basis for all the biogenetic regulations of the Council of Europe falls into the category of that ‘human dignity’ which every human being is entitled to, from the very first moments of prenatal development (Para. 19 Explanatory Report to the Convention for the protection of Human Rights and Dignity of the Human Being with regard to the Application of Biology and Medicine: Convention on Human Rights and Biomedicine; Para. 84 Case of Vo v. France; Kemp, Rendtorff, Johansen 2000; Andorno 2013; Barilan 2012). ‘Inherent dignity’, then, in the light of the provisions of The Universal Declaration of Human Rights as well as acts on the protection of human rights that came later, is the source of all concrete rights and freedoms. Reference, then, to this specific normative essence of humanity, with regard to the first moments of prenatal development, whilst even denying the human embryo concrete rights and any recognition of their freedoms, and even the unequivocal refusal to grant them the status of a ‘human’/‘person’/‘everyone’, bears all the hallmarks of a kind of revolution. Through this step, The Council of Europe’s system for the protection of human rights is undoubtedly significantly extended, which is a fact that should be very clearly pointed out.

The last of the objections regarding the nature of the interpretation of biogenetic provisions, in particular when in conflict with the axiological systems of The Convention on Human Rights and Biomedicine and The Convention for the Protection of Human Rights and Fundamental Freedoms, could indeed be justified if these systems remained, to even some extent, independent of one another. Yet this is not the case. The Convention on Human Rights and Biomedicine is directly connected to The Convention for the Protection of Human Rights and Fundamental Freedoms’ philosophy and system of values, and extends its provisions in the context of the use of biology and medicine. And thus, the axiological contradiction, and consequently the legal one, between the provisions of both treaties is, a priori, ruled out. This does not mean, however, that law enforcement bodies cannot have real difficulties in reconstructing the axiology and contents of The Convention on Human Rights and Biomedicine – carrying out its statutory interpretation in good faith and in accordance with the purpose and object of the treaty (as mentioned above), allows the interpretation of its norms which ensures the cohesion of the entire system.

In underlining the significance of this ‘revolution’ within The Council of Europe’s system for the protection of human rights through the introduction therein of the issue of biogenetics, it is worth remembering the transformation which, in this way, has taken place within this same model of human rights. The form in which human rights are currently encountered really exploded onto the scene after the Second World War, and became a response to the crimes of totalitarian regimes (Koba, Zydel 2009). These rights were intended to protect the individual, and possibly a specific group of individuals, against abuses of power. This point of view, underlining the verticality of human rights, for a long time dominated in Western societies. Over time, people also started to notice the transversal dimension of the applicable human rights on the relationships of the individual – the individual it must be, as a term – for any other subject is not part of the apparatus of State power (Piechowiak 1999). However, even then the relationship between the individual and the State seemed to be the natural and principal area of regulation. We can even risk the claim that in the above cases – in the workplace – biogenetic regulations lead to a total re-evaluation of these relationships. The relationships arising in the horizontal dimension are starting to play a leading role against which the relationships of a vertical nature are turning out to be of secondary importance.

The ‘shackles’ in which the biogenetic standards of the Council of Europe can be said to have to operate in are not, then, imposed upon power (the State), but above all, they hold back from individuals the opportunity to take part in specific research activities or procreative activities, or rather, perhaps, activities which are adjunct to procreation. And so, in the case of traditionally understood human rights, the line of conflict runs between the individual, or a group of individuals, and the State’s power, and so, relating to the specific problems of biogenetics, this line of conflict runs between the human being – understood as a *sui generis* subject of human rights, and another individual, or a specific group thereof – and people wishing to implement their own procreative plans, or researchers, patients or even the whole of society. Following this path of reasoning, it is important to note that until now the right to the development and freedom of research could, first and foremost, be recognised in terms of human rights, and only secondly to this as long as these do not violate the rights and freedoms of anyone else, but currently – due to the expansion of the scope of human rights or, most of all, the areas dealt with by their regulations – a competitor is growing in size: that of the freedoms of human beings in activities which could potentially violate their dignity and identity. With this freedom at the forefront of a system of values which is the foundation for The Council of Europe’s system for the protection of human rights, this freedom also represents its axiom and paradigm.

The shift which took place within The Council of Europe’s system for the protection of human rights as a result of the introduction therein of the biogenetic problem, should be considered as entirely legitimate. In the face of a kind of ‘inflation’ of human rights (Freeman 2002; Orned 2002), which, in the light of certain points of view should even be extended into the animal world (Cavalieri, Woollard 2004), the discourse on the various directions of the regulating of progress in the fields of biology and medicine seems to position the problem of human rights – both on a philosophical as well as a normative level – in its most appropriate location. If the idea is seriously treated that human rights are universal, natural and inalienable and belong to every human being regardless of any circumstance, then for activities and statute law and other decisions, etc., it is difficult, at this point in time, to imagine a more adequate subject for regulating than human biogenesis. The threats which appear with biomedical progress and the potential intervention in human beings’ existences who are in their prenatal stages of development concern the very essence of humanity. This is not only about what being a ‘human’/‘person’/‘everyone’ or ‘human being’ implies – it concerns every single human being. At this time, it should be pointed out that one of the allegations that has, for years, been voiced against human rights, was its clear lack of stating what or who is human, exactly, and whose rights and freedoms this was meant to refer to – as well as what humanity precisely means (Bała, Wielomski 2008). Due to the regulation of human biogenesis, this problem has become one of the central points in the debate.

Returning to the discourse on the scope of the subjects entitled to benefit from human rights moving into the area of the sources of the axiological doctrine means, moreover, that the problem of biogenetics and bioethics are both hard to assign to a specific group of rights and freedoms. Let us here use the metaphor put forward by René Samuel Cassin, explicitly referring to The Universal Declaration of Human Rights, but which also suits perfectly the description of other systems of protection. Cassin compared The Declaration to the portico of a Greek temple in which the foundation blocks constitute the principles of dignity, liberty, equality and brotherhood, the steps leading to the entry described in the seven paragraphs of the preamble and the same entry based on the four columns, including: 1. rights of the individual, 2. rights of the individual in civil and political society, 3. spiritual, public and political freedoms, and, 4. social, economic and cultural rights. The edifice of the temple was meant to be crowned by those obligations and limitations which enabled the realisation of social and international order, in which the rights and freedoms contained in The Declaration could be fully implemented (Zajadło 2006). The problems regulated in The Convention on Human Rights and Biomedicine, in the framework of the above image, can be qualified threefold.

Firstly, the least elegant way – and which, at the same time somewhat dilutes their essence – is the removal of individual provisions from between the columns of the temple. The second, reflecting their philosophical nature and essential meaning for human beings, and simultaneously underlining – at least in the case of human biogenesis – the lack of specific rights to which human individuals are entitled to in their prenatal stages of development, is for them to be written into the foundation blocks themselves. The third option is the building of an extra column.

Just as much as the third option seems the most appropriate in the context of the entirety of the bioethical regulations of the Council of Europe – the separation of the rights and freedoms threatened in the context of the development of biology and medicine may meet conditions of the validity – if not of logical partitioning – then at least of the typology, then so in the case of human biogenesis, indication in the foundation blocks should be considered appropriate. The protection of human beings in the initial stages of development, as mentioned above, is essentially accomplished without making them subjects for rights. This is based, in fact, on the dignity they are entitled to, which itself calls for respect. Of course, this dignity may constitute, as in the case of human beings, a concrete source of rights and freedoms. However, at the current stage of development in bioethical regulations, this has not yet been implemented. Remaining in the realm of allegory, one may well say that the source has not yet fully dried up. That said, this does not make the protection of unborn human beings illusory – wandering into the temple automatically means that we are crossing over into the ‘holy land’, in which the respect of the dignity of all human beings must be respected.

## Dignity as the basis of bioethical regulations

In the light of the above metaphor one can claim that one of the most distinctive and significant features of the biogenetic regulations of the Council of Europe is the clear acknowledgement of all human beings’ – from the first moments of their existence – as being worthy of human dignity (Para. 19 Explanatory Report to the Convention for the protection of Human Rights and Dignity of the Human Being with regard to the Application of Biology and Medicine: Convention on Human Rights and Biomedicine; Para. 84 Case of Vo v. France; Nowak 2011). Once again, let us underline the importance of the above step. Human dignity, after the tragic events of the Second World War, became the cornerstone of the ideology behind human rights. This is not a right which – in order to perform its functions – has to be laid down by the relevant authorities. There is something undoubtedly fundamental which is connected to the essence and existence of human beings and from whence emanate their basic rights and freedoms. We should note that these rights, along with their essence, are at the will of the powers that be, yet it is not these powers which give the individual that which truly confirms their existence. As an example to demonstrate this, in the very first sentence of the preamble of The Universal Declaration of Human Rights, the following appears: “Whereas recognition of the inherent dignity and of the equal and inalienable right of all members of the human family is the foundation of freedom, justice and peace in the world...”, and similar is the first sentence to the preamble of The International Covenant on Civil and Political Rights and The International Covenant on Economic, Social and Cultural Rights, as well as The Convention on the Rights of the Child. Moreover, in the International Covenants there appears the phrase that: “These rights derive from the inherent dignity of the human person”. The fact of the dependence of fundamental human rights on dignity was somewhat brilliantly reflected in the preamble to The Convention against Torture and Other Cruel, Inhuman or Degrading Treatment or Punishment: “…considering that, in accordance with the principles proclaimed in the Charter of the United Nations, recognition of the equal and inalienable rights of all members of the human family is the foundation of freedom, justice and peace in the world, recognizing that those rights derive from the inherent dignity of the human person…”.

The existence of dignity is, therefore, fully independent from the decisions of the authorities, and when faced with this phenomenon, may at most address this matter in some way or another. However, even if this rejected, this does not change anything. The existence of individuals deprived of dignity, as highlighted, amongst other things, by Personalism, is internally contradictory, but not only on a semantic level, but even violates the principle of consistency in its ontological approach (Ajdukiewicz 1985). At the same time, an individual’s existence – the being possessing human dignity – automatically implies the existence of a being entitled to human rights as well.

The structure of human dignity broadly appears in the soft law of the Council of Europe, and also in the Convention on Human Rights and Biomedicine and the Additional Protocols. In the Convention this also appears in the titles, preamble, and general provisions (Article 1 of the Biomedicine Convention). At the same time, its positioning and the methods of its use explicitly show that it fulfils two basic functions: on the one hand, it sets the aim, as established by the Member States of the Council of Europe – and possibly by the Convention on Human Rights and Biomedicine – and on the other hand, it becomes a directive in the light of which individual decisions must be interpreted (Para. 22 Explanatory Report to the Convention for the protection of Human Rights and Dignity of the Human Being with regard to the Application of Biology and Medicine: Convention on Human Rights and Biomedicine).

The exceptional significance of human dignity, as well as the implications that its recognition and respect might entail in the matter of human rights, in particular with regard to those problems which come about as a result of biomedical development, all require questions on its real status. The fact that this is the fundamental value for modern systems for the protection of human rights seems increasingly clear (Barak 2015; Montgomery 2005; Rosen 2012). Early prenatal development of the human being brings about, however, a number of objections – on the one hand, the legitimacy and on the other, the equitability of the extrapolation of the aforementioned value from this stage of embryogenesis (Grzegorczyk 1983).

Firstly, it should be noted that human dignity, as a phenomenon pertaining to an axiological reality, not a natural one, goes *beyond* purely biological existence. In fact, there is nothing physical which could be examined ‘under the looking-glass’ of scientists, yet which, in itself, constitutes ‘human dignity’, even when its abstract character is acknowledged – that is to say the a priori assumption that this dignity is not a substance, but rather a disease. Consequently, the acceptance of its existence in the face of the impossibility of prima facie evidence, with the help of either direct or even indirect observation, leads us to the conclusion that, in fact, this is actually a specific metaphysical construction, presenting itself in a more or less arbitrary fashion. To state that it is an inherent property of all human beings does not alter the fact that, in addition to the phenomenon of biological existence, we are still left with a transcendental reality. This problem comes into sharpest relief when seen in connection with human biogenesis – a juxtaposing and grounded analysis of human and animal embryos can, in no way, provide us with any differences in the field of axiology. The reasoning which ignores the gap between natural reality and moral reality is, therefore, inevitably exposed to the accusation of being a naturalistic fallacy. The extrapolation of the category of human dignity to the early stages of a human being’s prenatal development is, first and foremost, an operation carried out on an axiological level; thus, in a democratic society, the adoption of the principle of the ideological neutrality of the State requires a particularly solid justification.

Secondly, we should point out the difficulties surrounding the specific connotation of the term ‘human dignity’. Recognising that this is an inherent component of human existence does not bring with it any specific meaning, and therefore does not make of the being who possesses it the subject of any specific obligations. The same goes for the universality – and versatility – the inalienability, the non-gradability, the absoluteness, the permanence, and the equality of dignity. All of these things go some way to explaining what it is *like*, and not *what* it actually *is* (Picker 2007). In view of the above, the question arises of whether human dignity as such, is in fact, something different from the straightforward fact of belonging to the *Homo sapiens* species (Grzegorczyk 1983), to which we have learnt, over time, to attribute a particular moral importance. In consequence, dignity – from an ontic point of view – would cease to be an inherent feature of the nature of every human being, and would become at most, the most important – although maybe the result of a kind of ‘social contract’ – and the most specific act of will.

Thirdly, it is not clear what type of consequences one must tie in with having human dignity, beyond those which are indicated in legislature (Caulfield, Brownsword 2006). It would appear that the acknowledgement, by the subject of the international law in the specific area of human rights, even if human dignity is not indicated as the source of this, does in no way diminish from these rights. Furthermore, the claim that the versatile, universal, inalienable, permanent, and equal source of human rights is, itself, dignity, whilst simultaneously accepting – as a fact – the evolution which takes place within systems for the protection of human rights, may lead us to the conclusion that there are constant violations being committed in relation to this. It is barely worthy of mention at this juncture, that the law, by its very nature, always remains a step behind an ever-changing reality. The existence of specific acts usually precedes the development of the legal regulations relating to them. Consequently, what will tomorrow become a human right, today is, most likely, a violation of human dignity, and this will remain unchanged for as long as it takes the evolution of the systems for the protection of human rights to play out. Therefore there can never exist total protection. As an aside here, it is worth mentioning that this statement seems to shift human dignity out of the scope of what it currently *is,* to what it *should* be. Also in this way, paradoxically (from the critical position of the structure human beings’ dignity) we are lead to its affirmation, in a form which partly appears in systems for the protection of human rights – with the system of the Council of Europe at the fore of this – which should represent the aim of Member States (Pinker 2008).

The aforementioned reservations in this category of *in abstracto* human rights, and the responses given doubtlessly go beyond the domain of this particular piece of this artice. Due to the importance of the described construction for the biogenetic regulations of the Council of Europe, it is worth briefly referring back to them, and relativizing them to human biogenesis. Above all, one should note that the metaphysical character of human dignity does not detract from its importance in itself. In fact, the majority of values protected by law have just this kind of character. What is more, the whole legal system can be recognised as a metaphysical phenomenon. The reality of nature in itself does provide a causative reason for any legal system. It is the fact that human beings go beyond a purely physical reality that is important to acknowledge as the first step on the road to the creation of a culture, which is, amongst other things, a legal system. Accusations of a metaphysical nature aimed at the central element of a particular legal system in the cases in which the whole system already *has* this very nature, is, therefore, hard to see as legitimate.

In considering the legitimacy of the step of extrapolating human dignity to the stage of embryogenesis, it is necessary to take into account the fact that the human being in the early stages of prenatal development is not party to any relationships which actually bring people together – these relationships are only possible in postnatal stages of development. Such relationships providing sufficient reason for the acknowledgement of the parties’ inherent dignity, do not therefore become applicable to humans in their prenatal stages of development. The only ‘foothold’, so to speak, might therefore be the nature of the human being, whose foundation is his or her biological and genetic identity. Let us recall that, in accordance with the recommendations of the Parliamentary Assembly and the Council of Europe, the development of human life, which began in the instant the female’s egg was fertilised, is a continuous process (*Recommendation 1046 (1986)*, para. 5) and consequently any introduction of clear, non-arbitrary distinctions that could be recognised as morally or legally relevant, is therefore impossible. From the perspective of human embryogenesis having passed through to the next stage of prenatal development – the zygotes, morula, blastula, etc. – the human being, despite that fact that all this is of utmost importance to the full development of any particular, potential human being – nonetheless remains irrelevant in terms of identity, as this will be determined at the moment when the gametes are mixed (*Recommendation 1100 (1989)*, para. 7). This identity, which might as well be called human nature, not only maintains continuity in prenatal stages of development, but actually includes within it the whole life of the human being. At the same time, the early stages of embryogenesis appear to be the most vulnerable and open to modifications. If it is the case that human nature is to be protected in postnatal stages, when any opportunity for its modification is already, from a biological point of view quite dramatically limited, then surely protection is more necessary at those times when the developing human being is at its most ‘flexible’. Therefore, leaving humans being in their prenatal stages of development with no protection in the face of the current, advanced levels of science and medical techniques, may directly lead to a violation of the good which is at the very core of human civilisation and even humanity itself.

And so support, in an unexpected way, is, for the above-presented reasoning also backed up by arguments of a pragmatic nature which have been put forward by proponents for the unhampered development of biomedicine – and access to it. The basis of the hopes connected to the use of human embryos for purposes of science, therapy or diagnosis, is therefore embryos’ resemblance to people themselves. This similarity is based upon precisely the possession, common to both groups, of a nature of the exact same kind. Let us note the obvious truth that when human embryos do not possess a ‘human’ biological and genetic identity, the possibilities for their use for the aforementioned purposes, would surely not be so promising. For this reason, the specific moral and legal status of the human embryo shows itself to be immeasurably more controversial than the determining of the status of other cells or tissues. If we therefore acknowledge that human nature constitutes a value worth protecting, as systems for the protection of human rights clearly indicate, then the step of extending this protection into the prenatal phases of a human being’s life should be properly evaluated as being highly fitting. At the same time, it also seems appropriate to construct protection mechanisms around human dignity as well. What decides the unique position of every person is that which he or she shares with the unborn human being.

Considering dignity as a feature of all human beings (including persons as a sub-group of human beings), is also tied in with their specificity. This is the most general category, and without a doubt does not leave out any single member of the *Homo sapiens* species (para. 14 Explanatory Report to the Convention for the protection of Human Rights and Dignity of the Human Being with regard to the Application of Biology and Medicine: Convention on Human Rights and Biomedicine). This is also a category built upon that most basic of things and at the same time, universal in the species – its biological and genetic identity. Doubtlessly, this is why the creators of The Convention on Human Rights and Biomedicine decided to protect humanity on three different planes: an individual one, a social one and on the level of the species itself. The third of these emphasises the value of the individual as a member of the *Homo sapiens* species. What is human and pertaining to the species should be preserved for the good of future generations, and humanity as a whole. As noted by Francis Fukuyama, we wish to protect our complex nature, which has been shaped by evolution, against any of our own attempts at modifying it. We do not, however, wish to destroy the unity or the continuity of human nature along with those human rights which are based on it (Fukuyama 2004). It seems that the best means to achieve this goal is to bring the *whole* period of a human being’s life up onto the level of values mentioned above – fundamental, universal, versatile, inalienable and non-gradable values – which therefore amounts to recognising the dignity humans are entitled to.

The second of the allegations, in the context of human biogenesis considered normatively, also seems to lose some of its importance. The difficulty defining the nature of human dignity, in fact, cannot be translated into the way it functions within the framework of legal systems. From the point of view of the effectiveness of the system of human rights protection, the secondary question remains of whether this value possesses an ontological nature – it is inscribed in the human being but it also has a conventional nature – yet of far greater importance is the problem of its normative character. So if one assumes that this is the ‘only’ result of the agreement, then this agreement, given the role that human dignity plays in international law, must refer, then, to a specific axiom. From the moment the social contract is set in place, it will not be subject to discussion, and will be a superior value, a central, basic principle of systems protecting human rights: the highest, leading principle of a kind of normative order to things (Picker 2007).

Furthermore, it should be noted that ‘human dignity’ is not a category of meaningless content. In literature on the subject, pointed out everywhere are at least two sources which have left their mark on the modern form of the idea, and they are: Kantianism and Personalism (Lawer 2009). In the opinion of Immanuel Kant: “Everything has either a price or a dignity. Whatever has a price can be replaced by something else as its equivalent; on the other hand, whatever is above all price, and therefore admits of no equivalent, has a dignity” (Kant 1953). This sentence, in the context of the axiological sources of human rights, can be read as an unambiguous assigning of humans into the realm of beings not subject by their nature to any type of valuation. This implies also the irreducibility of humans to other categories which could make them the subjects of values, and therefore of things. For this reason, a human – in the opinion of the philosopher Königsberg – cannot be treated as a means in him−/herself in achieving specific – even their own – purposes, but must always be treated as a purpose in themselves.

From the above are derived two principal consequences: first and foremost, human dignity is synonymous with the non-reducibility of humans into categories which could serve as a means to achieving any particular goal. In connection with human biogenesis and the problems which are brought about by the development of biomedical science and its new techniques, it is the case that, inter alia, the embryo as a being entitled to human dignity can, in no circumstances, be subjected to total instrumentalization. Consequently, it cannot be used only for diagnostic, therapeutic or testing purposes. It can also not constitute the sole reason behind the realisation of any procreative plans. It must constitute its own supreme goal. Secondly, possessing human dignity is directly linked to the functioning of things on a moral level. Possessing human dignity means, therefore, that the existence of the individual is a subject of moral law. Moreover, in Kant’s opinion, law is not transcendental in relation to the individual, and comes from no external power, just as it is not anchored in any exterior object. As stated by the aforementioned philosopher of Königsberg: “The starry sky above me and the moral law within me” (Kant 2002). If it were any other way, it would cease to be the subject of moral norms and would become a slave to that which was at its source. Morality thus has its source in the individual, more specifically in the goodwill, which, in turn, is rooted in freedom. The individual therefore operates freely when he or she operates in accordance with dictates of reason which lie outside the vested interests of the individual, and what follows from this is not only desirable, but also a different form of external pressure – social requirements, divine injunctions, and so on. In such working, it so happens that the moral deed is the deed which should be fulfilled. Therefore, reason rediscovers the imperative which is that which should guide the conduct of the individual. Kant called this the *categorical imperative* as it indicates the functioning which is objectively necessary, and at the same time leaves aside all vested and external interests. “Act only according to that maxim whereby you can at the same time will that it should become a universal law without contradiction” (Kant 1953), and this is complemented – because of its purely formal nature – by The Formula of Humanity: “act in such a way that you treat humanity, whether in your own person or in the person of any other, never merely as a means to an end, but always at the same time as an end” (Kant 1953).

In accordance with Kant’s philosophy, morality is therefore established inside the human being, and is a manifestation of his freedom and will based on reason, which qualifies the dignity. Consequently, the categorical imperative, and The Formula of Humanity, actually exist objectively, to a certain degree. Despite the fact that the individual alone discovers these inside himself, he does not create them – they are universal and necessary principles, like the laws of mathematics.

The second line of thinking which has extensively influenced understanding, as well as the introduction of human dignity into acts relating to the protection of human rights, is Personalism. Supporters of this line of thinking acknowledge the person as the subject here, not only in a physical sense, but also in a moral one, and for this reason, the human is always ‘someone’ and not just ‘something’ (Wojtyła 1982). Being a human does not merely boil down to biological existence, but also to existing in a sphere of values. In other words, functioning in a sphere of values is a natural and essential part of human existence, and enshrined in humans’ essence. Consequently, human beings, due to their ontological structure, cannot be reduced to any other more general category. In such a way, the human would lose exactly that which defines it. It is precisely that which renders it impossible for human beings to be on the same level as an animal or the rest of nature, which defines their dignity, and *is* this dignity – even more – dignity itself is the embodiment of the value. And so, dignity is an absolute value and a constitutive part of the person, which does not require to be attached to anyone in particular in order to exist, nor to any society or external natural reality (Duchliński 2004; Mazurek 2001; Rodziński 1989).

The above presentation of dignity in which the human being is unequivocally placed within this sphere of values, means that being a human, necessarily, implies the existence of specific principles and rights. This was well expressed by Jacques Maritain – the brilliant French philosopher – whose views influenced, inter alia, the shape and nature of The Universal Declaration of Human Rights. “Human beings have rights just for the fact of being human beings, their own masters and perpetrators of their own actions, and as a result of which is not only a means, but a purpose in itself, and which should thus be treated. Human dignity means nothing if it does not necessarily imply that a person naturally has the right to be respected, and, as a subject of the law, has rights. There are certain things that a human is entitled to, just for the fact of being a human” (Maritain 1944). At the same time, Franciszek Janusz Mazurek wisely points out that the above rights do not so much come about from the dignity of human beings as (remembering that dignity is one of the constitutive elements of people) from that which lies within its ontic structure (Mazurek 2001).

The most essential of these rights to which human beings are entitled to – for the sole reason of being humans – is the right to be treated as a human being and as a person. According to Maritain, this right basically includes the whole group of rights which we call those rights that human beings possess, and which include:

- the right to exist and live a life corresponding to human dignity,
- the right to personal freedom and the freedom to choose one’s own direction in life,
- the right to personal development,
- the right to the freedom of conscience
- the right to choose one’s own beliefs to partake in the eventuality of marriage through free choice, and the right to have a family,
- the right to physical integrity,
- the right to the use of all of the above (Maritain 1944).

At this juncture, it is worth noting that Maritain devoted himself to the intellectual and moral revolution which requires that we restore the philosophy of true faith in human dignity and our rights thereto, in order to find the real source of this faith. Reading the preamble of The Charter of the United Nations, in which the following words appear: “We the peoples of the United Nations determined (…) to reaffirm faith in fundamental human rights, in the dignity and worth of the human person” or the preamble to The Universal Declaration of Human Rights, especially the following words: “Whereas recognition of the inherent dignity and of the equal and inalienable rights of all members of the human family is the foundation of freedom, justice and peace in the world, (…) Whereas the peoples of the United Nations have in the Charter reaffirmed their faith in fundamental human rights, in the dignity and worth of the human person, (…) Whereas a common understanding of these rights and freedoms is of the greatest importance (…)” – all this clearly indicates that this revolution has succeeded.

The above presentation of human dignity renders unfounded, to a great extent, the third of the above allegations relating to the above described construct. Along with the Kantian position, as well as that of Personalism, as presented above, there exists the possibility of a relatively simple removal of those rights to which the individual is entitled solely due to their belonging to humanity (Bayer 2004). Of course, in the case of Kantianism, especially in combination with Personalism, this operation (given the formal nature of the categorical imperative) does not reveal much to us. Nevertheless, working within this framework, The Formula of Humanity enables the formulation of a ban on the instrumentalization of the human being, therefore the treatment alone of whom becomes the only source for the realization of specific purposes. In the contexts of related doubts on human biogenesis in connection with the development of biomedicine, this ban – even without entitling the human embryo or foetus to specific rights, apart from that of the right to respect – when separated from any vested interests shows, itself to be of significant importance (Shell 2008). Theoretically, the general principle of respect for the dignity of every human being, in itself, could be sufficient in providing adequate protection against the negative effects of the development of biology and medicine. In practice, the interpretation of rights which operate on an axiological level alone is particularly difficult, and the results thereof are relatively easy to discount.

The analysis of the biogenetic standards of the Council of Europe, built around the principles of respect for the dignity of human beings, shows us that the potential for any huge protection is limited. Another competing good which is entitled to protection to a similar degree as that given to human dignity, is the progress of science and research. Its importance was underlined in a number of recommendations, as well as in The Convention on Human Rights and Biomedicine, which regulates this progress but does not stop it. Simultaneous protection of both values has caused the necessity to develop rules which allow the resolution of conflicts potentially arising between them. This principle was expressed, *expressis verbis*, in Article 2 of the Biomedicine Convention: “The interests and welfare of the human being shall prevail over the sole interest of society or science”. It is worthy of note that in the process of its formulation, the Kantian Formula of Humanity clearly takes precedence. Regarding the similarity of both principles, in particular the expression ‘the sole interest’, this is, in fact, the equivalent to the above expression: “Never merely as a means to an end, but always at the same time as an end”. In other words the concept was expressed in both principles that the wellbeing of human beings and humanity itself should constitute the most significant value in all this, and any action should be subordinate to this value. However, these actions may simultaneously bring about the realisation of other goals along the way.

The protection of the interest of society or science, speaking generally of people, means that human beings’ dignity in prenatal stages of development is, in practice, protected by those actions which clearly instrumentalise this and which international consensus has agreed upon. Activity of this nature, for example, the creation of human embryos for research, is prohibited by Article 18 of the Biomedicine Convention, as well as reproductive cloning being banned in Article 1, paragraph 1 of the PCHB. However, research on human embryos – which can doubtlessly be linked with this instrumentalization – lies outside this scope. Therefore, we can observe in biogenetic regulations a shift away from the metaphysical – whose expression is written into human dignity – and towards pragmatism, which allows specific procedures in the name of the interest of society or science, or even of specific people. For example, the consistent use of the category of human dignity in its Personalistic approach is employed by the Catholic Church in its unambiguous condemnation of artificial procreation. In the light of the case law of The European Court of Human Rights one can therefore claim that such restrictions could be read as violating human rights; we must also recall that the Biomedicine Convention, in fact, is a furthering of the provisions of the Convention for the Protection of Human Rights and Fundamental Freedoms. This shift in the direction of pragmatism provides us with, in this particular case, a rejection of the draft recommendation on artificial human procreation. This draft is an example of a very consistent and coherent internal application of the principle of the primacy of human dignity (CAHBI*, Draft recommendation on artificial human procreation* from 10th January, 1989). The principle, interpreted as was done by the CAHBI, limits certain things to too large a degree, such as people’s autonomy in making decisions pertaining to procreation.

This shift is most likely the effect of the hectic search for agreement on the most contentious issues, and at the same time – at least in the scope laid out in The Convention on Human Rights and Biomedicine – the most fundamental ones. A fitting example of this is the question of research on in vitro embryos, which, in countries in which it is allowed, can be performed upon ensuring the embryo ‘adequate protection’. This kind of ‘enigmatic’ wording demonstrates the descent down to the lowest common denominator, which, in fact – and within the framework of the protection of human life in its initial stages – does not have any real significance (Nys 2008). One should recall here that this is one of the reasons why Germany and Austria refused to sign The Convention on Human Rights and Biomedicine.

Contrary to the above, the authors of The Convention expressed their belief that this would be a good starting point in the creation of national regulations in the field of bioethics – and therefore biogenesis. It is in this way should the standards described in this work be seen. These need to lay out the absolute minimum in ensuring protection against these types of use of biomedical progress, which have internationally been deemed worthy of condemnation. The fact that they became the subject of consensus also gave one to believe that they would be widely accepted and respected. Similarly, and despite the reservations which were detailed on the biogenetic standards of the Council of Europe, it should be acknowledged that elevating them onto the level of the foundation of human dignity is a step in the right direction. What is extremely desirable, as well as doubtlessly valuable concerning progress in biomedicine, must happen within the boundaries not only of that which serves individual human beings, but also be an expression of humanity. It would appear that the recognition of the principles of the dignity of human beings constitutes a good starting point for the achievement of this goal (Andorno 2009).

## The identity of the human being – Biological reductionism or a new basis for metaphysics?

Article 1 of the Convention on Human Rights and Biomedicine, in relation to human beings, and alongside the protection of their dignity, expressly speaks of the protection of their identity as well, and the Steering Committee on Bioethics, in its explanatory report, explains that this protection also applies to the protection of the human species as a whole (Paras. 14 and 19 Explanatory Report to the Convention for the protection of Human Rights and Dignity of the Human Being with regard to the Application of Biology and Medicine: Convention on Human Rights and Biomedicine). This is how the creators of the Convention seem to point out that the roots of humanity are embedded in that which is common to all members of the *Homo sapiens* species – their characteristic biological structure, governed by the human genome (paras. 14 and 15 Explanatory Report to the Convention for the protection of Human Rights and Dignity of the Human Being with regard to the Application of Biology and Medicine: Convention on Human Rights and Biomedicine). Yet this approach is as much significant as it is risky. The risk associated with this (and which will be looked at later on in the article) is the danger of biological reductionism – making the individual nothing but a natural reality. In this light, human beings become – as stated by Julien Offray de La Mettrie – a machine (*l’homme machine*) which is merely carrying out a fixed programme, and who only differs from the rest of the animal world in his complexity (Tatarkiewicz 2001). In such a way it would be possible to set the human being’s existence on a par with that of other inferior species, and this, in itself, would not therefore carry with it any associated moral obligations. And in a similar way humans existences could be taken advantage of.

The threats from the biological reductionism of the bioethical standards of the Council of Europe, and in particular those of The Convention on Human Rights and Biomedicine, are rebutted by the above described category of dignity. Human dignity should therefore directly influence, or – in a weaker form – always be connected with the biological structure of *Homo sapiens*. In other words, the human genotype – which is responsible for humans belonging to this particular species and having all their individual features – becomes its own value, taking effect from the very first moments of existence. The thoughts of John Paul II expressed this clearly. In an address to the fourth, ‘Pro Vita’, Plenary Assembly of the Pontifical Academy, he stated that the human genome does not only have a biological dimension, but also comes with an anthropological dignity (John Paul II 1998).

It is noteworthy that the anthropological foundation, along with the dignity to which John Paul II was alluding, and despite a growth in the Catholic Church’s teachings of man being comprised of both spirit and body, in practice, lends perfect meaning to this form of anthropology adopted by the creators of The Convention on Human Rights and Biomedicine. And so the biological identity of human beings, along with their dignity, all constitutes an inseparable unity. The basis of metaphysics – so the construct of human dignity – is human beings’ biological structure, and never exists in isolation from the metaphysical component. For this reason, all intervention carried out on human beings – especially on the genome – should first and foremost take into account their wellbeing (Article 13, Biomedicine Convention). Therefore, any form of discrimination against a person on the grounds of his or her genetic heritage is prohibited (Article 11, Biomedicine Convention).

Others to speak of the human genome were Francis Fukuyama and Jürgen Habermas, inter alia. The first of these thinkers, seeking the source of that which constitutes the basis for the equal recognition and respect for all members of the *Homo sapiens* species, introduced into the discourse the ‘X factor’ notion. The *X factor* is the essence of humanity, and the most basic meaning behind being a human. According to Fukuyama, if all human beings are endowed with equal dignity, then the *X factor* will be their common feature (Fukuyama 2004). For Christians, this *X factor* arose from the Creator creating man ‘in his own image’, for Kant, it derived from the universal ability of people to make moral decisions. For Fukuyama, the *X factor* was a result of the complexity and complicated interactions which only ever take place within the sphere of human characteristics, such as moral choices, reasoning, and a wide array of emotions. Despite the fact that in specifically pointing out human characteristics, the above author makes reference to higher mental and intellectual functions, human nature remains for him the sum of the behaviour and characteristics typical to the human species, which are the result of genetics and *not* environment. The possession of a human genotype is therefore the natural starting point when defining what humanity is, or as well, what/who a human being is. Fukuyama did not herein state that an organism in possession of a human genotype automatically constitutes a human being; the definition he adopts emphasises the behaviour and characteristics typical to the *Homo sapiens* species, such as consciousness, emotions, the tendency for social life, or language. He stresses, however, the fact that the embryo already possesses the potential to shape itself into a fully-fledged human being. It can be assumed that this potential is characteristic for the genetic structure of the *Homo sapiens* species (Fukuyama 2004).

The ideas put forward in this article concerning the species and those related ethics from which originate legal regulations, are in line with the thinking of the herein oft-mentioned J. Habermas. In his opinion, the specificity of bioethical (particularly biogenetic) problems forces us to depart from models aimed at resolving ethical issues, based on the individual’s perspective. As underlined by postmodernism, individualism is not able to deal with those dilemmas which biomedical development brings with it. Consequently, the subjective vision of morality, which, in large part was supposed to constitute a strong point – i.e. the almost total autonomy of individuals – is not capable of coming up with a coherent regulatory system which could protect individuals from their instrumentalization. In the German philosopher’s opinion, the protection of the individual is to do with that which is ‘supraindividual’ and common to each autonomous human being: our self-knowledge of being a member of a species (Habermas 2003).

Habermas’s aim, like that of Fukuyama, is for the status quo of a modern society to be maintained within the boundaries it has itself designated (Habermas 2003). If, therefore, one of the bases of Western civilisation is a mutual recognition of the autonomy of the individuals which make it up (an example of which is the possibility of creating one’s own direction in life), then that which is the basis of this autonomy must be excluded from the scope of what is disposable. This something is once again the genetic structure of the individuals who belong to the *Homo sapiens* species, both in terms of the species, as well as the individual. The protection thereof therefore becomes a sine qua non, not only for the respect, but also for the existence of a being – at least in a socio-legal sense – whom we used to refer to as ‘man’. Habermas explicitly states that natural origins are a necessary condition in our being able to understand ourselves as the ‘authors’ of our own lives, as well as equal members of a moral community.

Reference to the biological and genetic basis of human beings, as the foundations for a relevant legal model of anthropology – despite the related threats – would appear to be of significance here. As mentioned, these threats are primarily associated with a reduction of the individual to the purely biological aspects of their existence. In this way – which is to say by reducing individuals to their specific biological features – it is easy to find strong reasons supporting the differentiation of individual human beings. In order to narrow the scope of our consideration down to the issue of biogenesis alone, one may point out that in the literature on the subject there is a widespread practice of indicating those characteristics of human beings which appear (or disappear) during the embryonic phase, and upon which the moral status thereof is defined, and therefore also legal status. For example, this characteristic could be: possessing this specific genotype, reaching the ‘primitive streak’ stage, the beginning of cerebral bioelectrical activity, reaching the stage of being able to live independently outside the mother’s body, and so on (Ramsey 1970; Ford 1988, Serra, Colombo 1998; Warnock 2003). Recognising any of the above as legally and morally relevant will lead to an automatic division of the scope of the term ‘human being in the prenatal stages of development’ into two categories, varying in status. By introducing other ‘levels’ to humanity, based on biology, we are able to construct a kind of ‘classification table’ covering the entire duration of human beings’ existences, and are able to grant these existences – according to the stage of development – specific legal statuses.

A weakness of the biological basis with regard to the normative construction may, in addition, be mix-ups which are not only mutually independent, but even incompatible with each other. David Hume – and in his footsteps, George Moore – noted that between the spheres of fact and obligation there can be no immediate transition. So, biological reality alone does not create normative reality. Therefore, the fact that human embryos possess a specific genetic identity does not provide us with any real basis upon which related obligations can be built. Supporters of this point of view underline the fact that, in consequence, the granting to individuals of a particular moral or legal status is closely tied in with possessing those specific features deemed worthy of protection (Machinek 2007).

The differentiation of biological and normative orders, despite a solid philosophical basis within the framework of biogenetic regulations, does not seem the best way to proceed. A more substantial paradox of the biotechnological revolution representing the next, and perhaps the most important, piece of evidence of humanity’s mastery and dominance over nature (i.e. the natural world) is the much emphasised biological aspect of human existence. The vision of Man as a being, in substance moving beyond the confines of his own natural reality, was presented by Descartes, and has been disqualified. In searching for a basis for humanity, for that which is most definitely human, Descartes stated that this basis is the soul. Attitudes towards human beings, like the one above, has resulted in the general acceptance of the discreditation of the body (Nawrot 2007). The body therefore became a secondary, subordinated ‘I’. The order of things which seemed natural, and was based on the concepts of ‘cogito’ and ‘esse’, was, for a long time, turned upside down. By moving humanity out of the realms of the natural was, of course, conducive to the glorification of specific human characteristics. And so man was able to become the ‘Crown of all Creation’. Separating the essence of humanity from biological existence has lead, at least on an intellectual level, to a situation in which (at least with regard to its intellectual plane) the abstract understanding of humans began to be absolutized – this was the cost that came with being human, seen as a physically existing being. Eduard Picker, amongst others, strongly condemns this process, noting that dignity – being separated from the substrate of the idea of humanity – finds itself outside the scope of any valuation, thus beyond relativization as well. Dignity is – in contrast to its vectors – absolute. Consequently, something here is being protected, which if separated from human beings, loses all meaning (Picker 2007).

Just as one may acknowledge that there are reasons which allow different ontological classifications of human beings at the initial stages of their development, then so their extrapolation on the grounds of a normative system – especially in the light of the development of biomedical science and its techniques – then any intervention undertaken on the human embryo, in particular its genetic structure, affects – directly and irreversibly – the identity of the person-to-be. In essence, this identity, from the moment it comes into being until the end of human existence, does not substantially alter (Para. 7, *Recommendation 1100 (1986)*).

It is worth noting that by pointing out this constancy of humans’ biological and genetic identity, the Council of Europe did not differentiate between the status of the human being in prenatal stages of development with the status of the person (Reuter 2000). The treatment which consisted of the indication of the implications of interventions carried out on the aforementioned category of human beings concerning people, and the construction of their basis on the application of the usefulness of the extrapolation of human dignity from all human beings, whose development leads to the emergence of people. As already pointed out, this particular step should be considered as perfectly apt, for four essential reasons. Firstly, indicating how human beings should be treated in the initial stages of their development does, in no way, determine their ontological status, and thus does not make legal regulations simply the next of many voices expressed in the sphere of the philosophical debate. The highlighting of the continuity of human beings’ development becomes reason enough for the inclusion, to a certain extent, of the whole species within this protection framework. If one accepts that human, in a normative sense, ‘arise’ with birth, then it is difficult to deny the influence on their essence and existence of activities carried out on the embryo or foetus from which humans grow. By constructing a conclusion on the shape of ‘proof by contradiction’, it is relatively easy to demonstrate that the unambiguous statement of the incomprehensibility of the category of human dignity in regard to the prenatal stages of a human being’s development during which its biological and genetic identity is determined, given the doubtless continuation of these into the individual’s latter stages of development, leads us into the realm of the absurd.

Secondly, moving on to biological and genetic identity as a kind of basis for human dignity (Dute 2005), the latter is connected to a particular individual’s existence, and moves into the metaphysical realm of individual biological frameworks. Anthropology constructed in this way does not render the human being something which it is *not* or *should not* be, which is to say some kind of abstract existence only found in an axiological sphere, or even in the world of Platonic ideas (Kowalczyk 2009). Human beings, despite their having been attributed unique values, are not made unreal by their being risen up onto a supranatural plane. Similarly, this does not reduce them to the typically biological aspects of existence either. So humans are not simply an immaterial idea, soul, spirit or ‘thought’ capable of existing independently of the body, yet are neither something which can be separated in essence from the rest of the natural world (Wójcik 2007). This idea, employing the typical linguistic apparatus of religious ideology, was very well expressed by the aforementioned J. Maritain amongst others, who, developing the philosophy of St. Thomas Aquinas, stated that the soul and the body are two substantial co-elements of one and the same being, one and the only reality – the human being (Maritain 1939). Of course, the Council of Europe’s legislation employs other categories, despite their meanings being extremely similar: human beings simultaneously possess the characteristics of biological and genetic identity, as well as dignity. In this way will the danger of the reductionism and the idealisation of the human being be limited.

Thirdly, the connection between biological and genetic identity with the metaphysics of dignity, explicitly places the human being – i.e. beings possessing a specific genetic structure – into the relatively well-defined and rich axiological system, which is the basis of the Council of Europe’s system for the protection of human rights. This value system, covering the ideals and principles which are the common heritage of the Council of Europe (Article 1, Statute of the Council of Europe), in the case of biolaw provisions, may provide valuable interpretive pointers which allow the reconstruction of the standards contained therein.

Fourthly, protecting what, for human beings, appears to be that which is the most fundamental thing of all, the described regulatory model leaves us with a relatively broad framework for biomedical development to function in. The established model regulating the biogenetic problem – above all the conditions therein to which the use of techniques of medically assisted procreation must correspond, and which unequivocally exclude from their scope only those actions which are teleologically oriented towards the objectivization of human beings – render human beings as merely a means in order to achieve goals which are unrelated to their best interests.

## Conclusions

All of the above seems to lead to the conclusion that over the last twenty years we were in fact dealing with a progressing “Copernican Revolution” in the field of biomedicine, which undoubtedly had positive effects. First of all, the introduction of the “human being” construct into the orbit of legal regulations enabled developing a human rights protection system, and thus increased its effectiveness. With just this step, which unequivocally banned the instrumentalization of the human being, the organizers of the Biomedicine Convention created a legal instrument that responds to the many threats to human dignity that were valid twenty years ago.

The essence of the revolution mentioned in the title, however, is not limited to the above. Basing legal regulations on well-defined anthropology makes it possible to construct an abstract but legally valid model of a human being. Based on the model that I tried to outline in this paper, future legal policy can be shaped and rules regarding threats resulting from scientific and technological progress in the field of biomedicine, including those we are currently unable to predict, can be defined. And this precise element – the intentional elevation of anthropology to the legal level – seems to be the most important achievement of the Council of Europe in connection with human rights protection in the context of the development of biology and medicine.
